# Prevalence and Predictors of Overweight and Obesity among Young Children in the Children’s Healthy Living Study on Guam

**DOI:** 10.3390/nu12092527

**Published:** 2020-08-20

**Authors:** Rachael T. Leon Guerrero, L. Robert Barber, Tanisha F. Aflague, Yvette C. Paulino, Margaret P. Hattori-Uchima, Mark Acosta, Lynne R. Wilkens, Rachel Novotny

**Affiliations:** 1 Office of Research & Sponsored Programs, University of Guam, Mangilao, Guam 96923, USA; bbarber@triton.uog.edu (L.R.B.); taflague@triton.uog.edu (T.F.A.); paulinoy@triton.uog.edu (Y.C.P.); muchima@triton.uog.edu (M.P.H.-U.); macosta@triton.uog.edu (M.A.); 2 University of Hawaii Cancer Center, Honolulu, HI 96813, USA; lynne@cc.hawaii.edu; 3 College of Tropical Agriculture and Human Resources, University of Hawaii At Manoa, Honolulu, HI 96822, USA; novotny@hawaii.edu

**Keywords:** child obesity, Guam, Children’s Healthy Living (CHL), islander, Pacific, Micronesia

## Abstract

This study is part of the Children’s Healthy Living program in U.S. Affiliated Pacific region. The objectives were to estimate overweight and obesity (OWOB) prevalence and identify possible related risk factors among ethnic groups in Guam. In 2013, 865 children (2–8 years) were recruited via community-based sampling from select communities in Guam. Children’s demographic and health behavior information; dietary intake; and anthropometric measurements were collected. Logistic regression, odds ratio, t-tests, and chi-square tests were used to determine differences and assess covariates of OWOB. The results indicate that 58% of children were living below the poverty level, 80% were receiving food assistance, and 51% experienced food insecurity. The majority of children surveyed did not meet recommendations for: sleep duration (59.6%), sedentary screen-time (83.11%), or fruit (58.7%) and vegetable (99.1%) intake, and consumed sugar sweetened beverages (SSB) (73.7%). OWOB affected 27.4% of children. Children affected by OWOB in this study were statistically more likely (*p* = 0.042) to suffer from sleep disturbances (*p* = 0.042) and consume marginally higher amounts (*p* value = 0.07) of SSB compared to children with healthy weight. Among Other Micronesians, children from families who considered themselves ‘integrated’ into the culture were 2.05 (CI 0.81–5.20) times more likely to be affected by OWOB. In conclusion, the OWOB prevalence among 2–8-year-olds in Guam was 27.4%; and compared with healthy weight children, children with OWOB were more likely to have educated caregivers and consume more SSBs. Results provide a basis for health promotion and obesity prevention guidance for children in Guam.

## 1. Introduction

Childhood overweight and obesity (OWOB) is a global epidemic affecting many countries [1,2,3,4,5], including the United States (US) and the US Affiliated Pacific region (USAP). In the US a high prevalence of OWOB among racial/ethnic minority groups for children 2–19 years is reported, specifically Non-Hispanic black (19.5%) and Hispanic (21.9%), yet Native Hawaiian or Other Pacific Islanders are not included [1,2]. Novotny and colleagues conducted a systematic review of childhood OWOB in the USAP region and estimated that the prevalence of OWOB for children 2–8 years was 21%, and that the prevalence increased to 39% by age 8 [6]. Similarly, the proportion of obese children increased from 10% at age 2 years to 23% at age 8 years, with the highest prevalence of obesity in both Guam and American Samoa [6]. The systematic review found that in Guam, OWOB prevalence (39%) among children ages 3 to 5 years [6] exceeded the US national average (23%) for children aged 2 to 5 years [1,2].

Guam is a U.S. Territory located in the northwestern Pacific Ocean, approximately 3700 miles west of Hawaii, 6000 miles west of California, and 1300 miles southeast of Japan. CHamorus are the original inhabitants of Guam [7,8], and are typically grouped with other Pacific Islanders. However, the current population of Guam is characterized by substantial ethnic variation [9]: 37% CHamoru, 26% Filipino, 12% other Pacific Islander, 11% other ethnicity, 7% White, and 7% other Asian. This ethnic diversity evolved through centuries of colonization and migration that continues today [10] and may modify the burden and predictors of OWOB among CHamorus. Factors related to OWOB are sleep, physical activity, psychosocial, life course exposure, SES, and diet [11,12] that for many racial/ethnic groups in Guam is influenced by this history [12,13]. OWOB prevalence among adults is higher among CHamorus compared to other ethnic groups on Guam [11]. In a study among adults in the two largest ethnic populations in Guam then (2008) and now, CHamorus and Filipinos, there was a significant difference in energy density of diets between the two groups, where CHamorus had a higher energy and added sugar intake [11]. In 2009 and 2010, prevalence of OWOB was highest among CHamorus followed by Other Micronesians reported in one of the first reports to disaggregate BRFSS data by ethnicity [14].

Children who are overweight or obese have a higher risk of being overweight or obese adults [15] with an increased risk of chronic diseases later in life [16]. However, there is limited information about young children in Guam, and data are needed to understand the burden of and determine the appropriate intervention for childhood OWOB. Therefore, the purpose of this study is to estimate the prevalence of OWOB among children in Guam and identify demographic or other risk factors targeted by the Children’s Healthy Living (CHL) program for overweight and obesity in CHamoru children.

## 2. Methods

The Children’s Healthy Living Program for Remote Underserved Minority Populations in the Pacific Region (CHL) program is a partnership of universities and local organizations across the remote USAP (Alaska, American Samoa, Commonwealth of the Northern Marianas, Federated States of Micronesia, Republic of Marshall Islands, Republic of Palau, Hawaii, and Guam) working to prevent childhood obesity in the Pacific. Detailed information on the study design of the CHL program can be found elsewhere [17,18]. Briefly, approximately 900 children (2–8 years) and their parents/caregivers from each participating USAP jurisdiction were recruited to participate in the study at both baseline and 24-month follow-up. On Guam, a total of 865 children were recruited at baseline (2013) and 696 children were recruited from the same communities at 24-month follow-up. This paper will focus on baseline data of the 865 children on Guam recruited from communities (Agana Heights, Sinajana, Agat, Santa Rita, Yigo, Yona, Talafofo, and Dededo) selected due to their size, representation of indigenous (CHamoru) residents, and isolation and cohesiveness (for purpose of intervention) [17]. Behavioral targets of the CHL program were increasing sleep, water intake, fruit and vegetable intake, physical activity and decreasing sugar sweetened beverage intake and sedentary behavior [17].

Child participants were recruited primarily at early childhood education centers (e.g., Guam Head Start program, daycares, and public elementary schools) and community-based settings (e.g., municipal centers and public housing areas) on Guam. Data collection occurred over a minimum of two visits at these recruitment sites. Further details on recruitment is described elsewhere [17,18]. Child participants with parents’ consent and who provided assent were assessed.

At the first visit, parents/caregivers completed surveys on demographic information (i.e., child age, place of birth, race/ethnicity, and sex; household composition and food security; parent/caregiver educational level and income); cultural identity of the parent/caregiver; and general health status, early life feeding behaviors, and sedentary screen-time and sleep behavior germane to the child. They also received instructions for completing a two-day food and activity log at home during the week. Sleep quality was measured with the Tayside Children’s Sleep Questionnaire (TCSQ) [19] and sleep duration [20] was reported by the parent/caregiver as hours asleep at night and during naps. Other surveys were adapted from previous studies [17] with some local terminology added for participant clarity.

Detailed information was collected in CHL on race/ethnic groups, allowing for multiple groups to be selected; classification prioritized ethnic groups indigenous to the jurisdiction. On Guam, the indigenous ethnic group is CHamoru [7,8]. If more than one race/ethnicity was indicated for the child and CHamoru was selected, the child was categorized as CHamoru. If a child identified as not CHamoru and as one of the other Micronesian Pacific Island ethnic groups, such as Chuukese, Palauan, Yapese, Carolinian, Marshallese, or Kosraean, that child was categorized as ‘Other Micronesian’, because the only other Pacific Island ethnic groups identified in the Guam sample were from Micronesia and not Polynesia (e.g., Native Hawaiians or Samoans). These other Micronesian Pacific Islander groups were grouped together as their numbers were small and they were all from neighboring Micronesian islands.

Food insecurity was determined by asking one question from the US Department of Agriculture’s Core Food Security Module: “In the past 12 months how often does money for food run out by the end of the month?” (never, seldom, sometimes, most times, always, don’t know, or no response [21]. In this study, options of don’t know or no response were treated as missing values. Household food insecurity was considered present if the respondent chose sometimes, most times, or always. Parent’s cultural identity, or “acculturation,” was determined using their responses reported on a cultural affiliation questionnaire, which assesses one of four modes of acculturation, from two cultural identity subscales (i.e., respondent’s ethnic group and the US): traditional (high ethnic and low US subscale scores), integrated (high ethnic and high US subscale scores), assimilated (low ethnic and high US subscale scores), or marginalized (low ethnic and low US scores) [22]. The same scoring system was used as described by Kaholokula and others [22].

After providing assent, each child was measured for weight, height, and waist circumference (WC) by trained and standardized research staff [23] and described in detail elsewhere [17]. These measures were used to compute Body Mass Index (BMI) [weight (kg)/height(m)^2^], WC (cm) to height (cm) ratios, and subsequent BMI z-score, BMI-for-age-percentiles, and waist circumference- for-age percentiles [24,25]. BMI percentiles and z-scores were calculated according to CDC reference data [26] and BMI categories were assigned accordingly: underweight (<5th percentile), healthy weight (5th–84th percentile), overweight (85th–94th percentile), and obese (≥95th percentile). Children were considered to have OWOB if their BMI percentile was greater than the 85th percentile for age and sex. Cutoff values for biologically implausible values defined as <−5 or >4 standard deviations (SD) for height-for-age z-score and <−4 or >8 SD for BMI z-score (according to CDC reference data) were removed from the analysis. Child participants were considered to have abdominal obesity if their WC was greater than the International Diabetes Federation [27] cut-point.

Fruit, vegetable, and beverage intake were estimated using data recorded in the child’s two-day food logs reported by the child’s parent/caregiver and data collection methods are described in detail elsewhere [17]. Parents/caregivers were asked to complete the food log of everything their children ate or drank for two randomly assigned non-consecutive days, which included weekdays and weekend days, between visit one and two, approximately 6 days apart. Assignment of recording days was based on the day of the child’s first visit (Monday–Saturday). Parents/caregivers were instructed in record keeping techniques with the aid of food models, service ware, and measuring utensils. During visit two, research staff reviewed the food log with the parents/caregivers (e.g., for completeness of food entries, portion size estimation, food preparation methods, and/or accuracy of recording data). Trained staff entered the food log data into the Pacific Tracker3 (PacTrac3), which includes a food composition database developed in collaboration with the University of Hawaii Cancer Center for use in the Pacific region [28,29,30]. For this study, PacTrac3 data were used to classify beverage type (i.e., sugar-sweetened beverages (SSB), milk, or water), fruit, and vegetable, as well as, calculate intake (e.g., cups/day, grams/day). All soft drinks, fruit drinks (fruit flavored or containing less than 100% juice), sports drinks, energy drinks, sweetened tea, and sweetened coffee reported were classified as SSB. Milk intake in cups was calculated for food log entries and included all fluid milk (cow and/or goat), chocolate milk, lactose-reduced milk, lactose-free milk, filled milk, dry milk, and evaporated milk. Fruit, vegetable, milk, water, and SSB intake per day was adjusted for within person variance and then averaged over the two days of records, weighted for weekday and weekend days.

Compensation for study participation was provided at visits one and two [17]. The CHL program was funded by the United States Department of Agriculture (USDA), Agriculture and Food Research Initiative. Ethical approval for this project was granted by both the University of Guam Committee on Human Research Subjects (IRB) (CHRS#12-74) and the University of Hawaii Institutional Review Board (CHS#18915); written consent was given by all parents and oral assent was given by all child participants prior to their inclusion, in accordance with the Declaration of Helsinki.

Statistical analyses were performed using IBM SPSS Statistics version 26 (IBM Corporation, Armonk, NY, USA). Data analysis included the calculation of percentages for ordinal and nominal data, and means and standard errors for the interval and continuous data. *t*-tests and chi-square tests were used to test for differences in continuous and categorical variables, respectively, between BMI groups and ethnic groups. Binary logistic regression models of OWOB assessed its relationship with several potential covariates, adjusted for sex, age, and ethnicity and with the variance corrected for clustering of children within communities. Odds ratios and 95% CI were the primary statistics reported from the models. ORs were calculated for each of the following child factors from all ethnic groups combined and in CHamoru and Other Micronesian children separately: child age, sex, ever breastfed, sleep, sedentary screen-time, vegetable intake, fruit intake, water intake, and SSB intake; and parent/caregiver education, acculturation and marital status; and household income, food insecurity, food assistance, and household size. Does-response was assessed using a trend variable assigned consecutive integers (1, 2, …) to each ordered category. These factors were selected among demographic and lifestyle-related variables because they have been reported to be risk factors for OWOB among children [17,25,31,32] and/or were behaviors of interest for the CHL Program. *p* values < 0.05 were considered statistically significant, whereas *p* values of 0.05 to 0.10 were described as borderline significant.

## 3. Results

### 3.1. Demographics

The descriptive characteristics of child participants at baseline are summarized by ethnicity in Table 1. The average age of child participants was 5.79 years (sem 0.062). The majority of child participants were male (51.7%), 2–5 years old (53.8%), CHamoru ethnicity (64.8%), born on Guam (84.5%), from households where parents were not married (60.7%) or there were 3 or more children (76.2%), and considered as being ‘integrated’ (79.9%) as reported by parent/caregiver.

Overall, more than half of participating children (57.9%) came from families whose annual income was less than $20,000, which was slightly below the poverty level for a 3-person household in 2015 [33]. A significantly higher (*p* < 0.001) proportion of Other Micronesian children (82.9%) came from families whose annual income was less than $20,000 compared to CHamoru (54.2%), Filipino (43.24%) and Other (40%) children. Parents of Filipino children reported significantly higher (*p* < 0.001) education attainment levels compared to both CHamoru and Other Micronesians.

### 3.2. OWOB Prevalence

OWOB prevalence among child participants in this study is 27.4%. A significantly (*p* = 0.03) higher proportion of Other Micronesian children were affected by obesity (BMI ≥ 95th percentile) compared to CHamoru children (18.3% versus 11.0%) (Table 1).

Table 2 shows child participant characteristics by weight status for all ethnic groups combined, and for CHamorus and Other Micronesians separately. Children affected by OWOB reported statistically higher body size parameters as measured by height, weight, WC, BMI, and BMI z-scores, compared to healthy weight children. All children with abdominal obesity, as defined by the International Diabetes Federation [27], were OWOB. Among children in the OWOB weight status, 33.8% had abdominal obesity by this cut-point. It is worth noting that the International Diabetes Federation cut-point is for older children (6–10 years old) and is limited in identifying younger children (2–5 years) with abdominal obesity.

There were no significant differences in the presence of OWOB among children by sex, ethnicity, family income, food insecurity, food assistance, acculturation, screen-time, sleep duration, or intake of fruits, vegetables, water, or milk. OWOB children consumed marginally higher amounts (*p* value = 0.07) of SSB compared to healthy weight children. There were significant differences in the presence of OWOB among children by certain demographic characteristics, such as parent/caregiver education level, parent/caregiver marital status, and child birthplace (Table 2).

To explore further the relationship between the behavioral factors of interest and OWOB, we calculated ORs separately for CHamorus and Other Micronesians, the two largest ethnic groups in the study (Table 3). Among CHamoru children, those who were older (6–8 years) were 1.63 times more likely to be affected by OWOB than younger children (2–5 years). The highest prevalence of OWOB was seen among Filipino children (33.8%), followed by Other Micronesian children (28.9%), and CHamoru children (26.0%). OWOB was also more likely to occur among children ages 6–8 years (30.4%) versus children between the ages of 2–5 years (24.7%). No significant associations were found except that association between OWOB and SSB intake, and OWOB and parent education level.

### 3.3. Nutrition Factors

The majority of child participants (80.4%) were receiving some sort of food assistance, including USDA Supplemental Nutrition Assistance Program (SNAP) (67.8%), USDA Supplemental Nutrition Assistance Program for Women, Infants, and Children (WIC) (31.8%), free or reduced school lunch/breakfast (28.8%), and/or assistance from a local food bank (11.7%). Food insecurity was also prevalent as 51.5% of participants reported that food money ran out at the end of the month either always/most times (16.8%) or sometimes (34.7%). Significantly more (*p* < 0.001) Other Micronesian child participants (74.9%) reported that food money ran out at least sometimes compared to CHamoru (42.8%), Filipino (53.7%) and Other (50%) children.

As for OWOB risk factors and CHL related nutrition behaviors, majority of children did not meet recommendations for fruit (58.7%) and vegetable (99.1%) intake and consumed SSB (73.7%). Fruit and vegetable intake did not differ significantly by ethnicity (Table 1) or by the presence of OWOB (Table 2). Mean intake of vegetables was low (0.61 c/d) and only 6 children surveyed (0.09%) consumed the recommended amount of vegetables daily, which equates to ≥1 cup equivalent for children 2 years old and ≥1.5 cup equivalent for children 3–8 years old [34,35,36]. Mean intake of fruits was somewhat better (0.88 c/d) and more children (41.3%) reported consuming the recommended amount of fruits daily, which equates to ≥1.5 cup equivalent for children 2–8 years old [34].

When asked if the child participant was ever breastfed as a baby, about two-thirds (67%) of all parents/caregivers responded yes. Only 13.1% of children were exclusively breastfed (not shown in table), while 54.2% of children surveyed were fed both breastmilk and infant formula as an infant. CHamoru children (21.32) reported being significantly less likely to be ever breastfed compared to Other Micronesian children (37.2%). Of those children who were ever breastfed, Other Micronesian children were weaned at a significantly older age (12.48 m) compared to children from other ethnic groups. Breastfeeding during infancy, whether exclusive or mixed with infant formula feeding did not differ by the presence of OWOB. Age at weaning also did not differ by presence of OWOB. Among those who were exclusively breastfed, mean age at weaning was significantly higher compared to mixed fed infants (19.27 ± 1.19 m versus 7.56 ± 0.04 m). Risk of OWOB increased significantly with SSB intake. The relationship between presence of OWOB and increasing SSB intake was not monotonic, the ORs (95% CI) for SSB intake for the tertile groups for consumers compared to those with zero intake of SSB were 2.06 (1.02–4.16), 1.50 (0.90–2.48), and 1.82 (1.11–3.01). Only 25% of children surveyed consumed the recommended amount of “zero intake” of SSB [37], and one-third of child participants consumed at least 1.1 cups of SSB per day. Correlation analysis showed that SSB intake was positively and significantly associated with BMI and waist circumference. Among CHamoru children, the relationship between SSB intake and body size was stronger, as SSB intake among CHamoru children was positively and significantly associated with BMI (*r* = 0.18, *p* = 0.0002), waist circumference (*r* = 0.16, *p* = 0.0007), and BMI z-score (*r* = 0.11, *p* = 0.023). In Table 3, although SSB intake was not significantly associated with OWOB for Other Micronesian children, it was significant for CHamoru children. The relationship again was not monotonic, the ORs (95% CI) for SSB intake for the tertile groups of consumers compared to those with zero intake of SSB were 3.15 (1.1–9.08), 2.84 (1.28–6.30) and 3.19 (1.46–6.95).

### 3.4. Sleep and Screen-Time

Less than half of all children surveyed (40.4%) met the sleep duration recommendations [20]; those recommendations being at least 8–11 h/day for children under the age of 5 years, and 8–10 h per day for children 5 years and older. Sleep duration did not differ by the presence of OWOB, yet we observed TCSQ sleep quality scores (Table 2) were significantly lower (*p* = 0.0421) among children affected by OWOB compared to children with healthy weight (5.59 ± 0.34 versus 6.55 ± 0.26, respectively), meaning that children with OWOB were statistically more likely to suffer from sleep disturbances. Only 30.58% of Other Micronesian children met sleep recommendations (Table 1), which was significantly (*p* = 0.014) less than children from other ethnic groups. Other Micronesian children reported TCSQ sleep quality scores that were lower (*p* = 0.056) compared to children from the other ethnic groups.

The recommended daily screen-time for children is less than 2 h per day [38]. Only one sixth of the children surveyed (16.9%) met this recommendation and meeting screen-time recommendations did not differ between healthy weight children and children affected by OWOB. Average screen-time was slightly more than 5 h per day. When comparisons were made across four ethnic groups, there was a significant difference (*p* = 0.007) in the proportion of children that met the screen-time recommendation: CHamorus (16.7%), Other Micronesian (22.5%), Filipinos (4.6%), and Other (0%). There was also a significant difference in the average daily screen-time among the ethnic groups: CHamorus (5.13 h), Other Micronesians (5.45 h), Filipinos (6.24 h), and Other (3.59 h) (Table 1).

### 3.5. Acculturation

The majority of study participants (80%) considered their cultural affiliation as integrated, meaning a bicultural orientation where the respondent retains their ethnic cultural identity at the same time moving to join the dominant society (i.e., the US) [22,39]. About 15% of both CHamorus and Other Micronesians considered their cultural affiliation traditional, where the respondent retains their ethnic identity and does not recognize or identify with cultural characteristics of the US [22,39]. The Other Micronesians (6.1%) were most likely of all ethnic groups represented in this study to report having marginalized cultural affiliation, meaning the respondent does not maintain his/her ethnic cultural characteristics and excludes or withdraws him/herself from the dominant society (i.e., the U.S.) [22,39]. Acculturation did not differ by the presence of OWOB (Table 2). However, we observed that among Other Micronesians, children from families who considered their cultural affiliation as integrated into the culture were 2.05 times more likely to be affected by OWOB (CI 0.81–5.20) (Table 3).

## 4. Discussion

Pacific Islanders were one of the fastest growing racial/ethnic groups in the US in 2000 to 2010 [40], but little is known about OWOB for young children (under 11 year) in the Pacific region [41]. The current prevalence of OWOB in the US is 25% among children between 2–5 years and 32.8% among children between 6–8 years [42]. Findings in this current study on Guam were consistent with previous reports [43] and similar to the US. Unlike previous reports, this study revealed ethnic differences in OWOB presence among children in Guam. Filipino and Other Micronesian had the highest OWOB (33%) and obesity (18%) prevalence compared to other children in Guam.

Some recent studies have shown that obesity among various Pacific Islander groups, such as Samoans, may be linked to specific genes influencing adiposity [40,41,44]; and Pacific Islanders of Polynesian, Micronesian, and Melanesian origin may all carry certain alleles associated with higher body weight, BMI, and risk of obesity [41,45]. Waist circumference-to-height ratio (WHR) and WC have been used as markers of body adiposity in adults and, in children and adolescents (8–18 years), WHR was determined to be better at predicting adiposity [46,47,48]. One third of this study’s participants (33.8%) were observed to have both, abdominal obesity and OWOB. On the other hand, healthy weight children did not have abdominal obesity (0%). This aligns with other studies that found adiposity indicators, like WC, were associated with being overweight [47]. This is alarming as the location and distribution of body fat are associated with cardiovascular and other disease risks that is likely to continue into adulthood [49,50,51].

Given the steady rise in obesity over the past few decades on a global scale, environmental (macro and micro) factors promoting obesity are a more likely predominant explanation for racial/ethnic disparities in childhood obesity [52], such as the increase in obesity seen on Guam and other islands in the Pacific. A majority of children in this study exhibited several behaviors that are obesity risk factors such as low intake of fruits and vegetables, high intake of SSB, low sleep duration, and high amounts of sedentary screen-time [52].

From the micro-environment of children, which includes their families and households, in this study, the majority of child participants came from households where their parent/caregivers were unmarried (60.7%) and with at least three or more children (76.2%). Those two factors, compounded by the fact that most of the parents in this study also reported low annual incomes, can lead to an unfavorable home environment. Research shows that family and home environments, whether directly or indirectly, may contribute to the development of child OWOB [53,54,55], possibly through maternal depression and stress. Further study and more specific questions regarding parent/caregiver psychological health is needed to determine impact of family environment on the presence of OWOB among children in Guam.

Surprisingly, 59.65% of the children surveyed did not meet the recommended sleep duration for their age. Research indicates that short sleep duration puts children at risk for obesity and other metabolic, cardiovascular, and behavior disorders [56,57,58]. This study did not find sleep duration to differ significantly between children with OWOB and those with healthy weight, possibly due to misinterpretation of the question on sleep duration. Given some of the very low reported levels of sleep hours per day for some of the children and limited English comprehension of the islander parents/caregivers, it is possible that nap times during the day may have been the reported time, rather than sleep over the 24-h period. Future studies should separate questions for nap time sleep from night sleep in determining USAP children’s sleep duration and include more qualitative non-subjective measurements for sleep duration. In terms of sleep quality, children affected by OWOB in this study were statistically more likely to suffer from sleep disturbances. This aligns with the research linking poor sleep quality with OWOB and an increased risk for cognitive and behavioral problems [56,57,58]. Research also links reduced sleep quality with high SSB intake, and in bed screen-time [59].

The proportion of children exceeding screen-time recommendation in this study (83%) was comparable to the 87% reported among a prospective birth cohort of children born in New York led by researchers from the NIH Eunice Kennedy Shriver National Institute of Child Health and Human Development [60]; however, the birth cohort included children below two years of age, which is an age group not represented in the Guam study. Additionally, while screen-time increased with preschool age in the birth cohort, the total hours decreased to below the recommended two hours by 7–8 years old possibly due to school activities [60].

Screen-time has been shown to be associated with other behaviors, especially sleep. Twenge and colleagues [61] found an inverse association, increased screen-time and reduced sleep duration among a population-based sample of 0–17 year-olds in the United States [61]. In the current study, the order of screen-time, from highest to lowest duration, by ethnic group was Filipino (6.24 h), Other Micronesians (5.45 h), CHamorus (5.13 h), and Other (3.59 h). Interestingly, this order was reversed with the TCSQ sleep quality score, where Filipinos reported the highest quality sleep (5.23), and Others reported the lowest quality (8.0), but the pattern was not the same with sleep duration. Researchers are beginning to better understand the impact of screen-time on sleep behaviors. For example, Guerrero and colleagues [62] found a relationship between increased screen-time and increased social problems (e.g., rule-breaking, social problems, aggressive behavior, and thought problems), and the relationship was mediated by sleep duration (every hour increase in sleep = 8.8% to 16.6% decrease in problem behaviors) among 9–10 year-old in the Adolescent Brain Cognitive Development study [63]. Behavioral problems, although complex, are real issues plaguing children and should be considered when building wellness models for healthy families and healthy communities. Further studies on screen-time among children in Guam are warranted.

Childhood behavioral problems have been linked to racial socialization and cultural resilience [64]. Similarly, cultural affiliation, or low acculturation (i.e., traditional), is associated with lower rates of obesity and sedentary behaviors in children from migrant populations [39]. Although CHamorus in this study are not likely migrants, the long history of colonization in Guam lends to multidimensional acculturation for CHamorus and Guamanians alike in the presence of the colonial society (i.e., the US). This considered, CHamoru children had the highest proportion of parents identifying as traditional and the lowest rates of obesity compared to other ethnic groups in this study. Additionally, Filipino children identified most as integrated had the highest average screen-time (hr/d) compared to other ethnic groups. These findings align with previous studies that have found traditional or low acculturation to be protective against obesity and related risk factors [39,65,66]. A greater acculturation (i.e., assimilation) has also shown to be a predictor of lower fruit and vegetable intake among adult populations [67,68]. Therefore, obesity interventions or health programs in Guam should include cultural values and practices to maintain or reinforce healthy cultural behaviors unique to each and shared across ethnic group(s).

Both fruit and vegetable consumption among child participants in this study were low and failed to meet the recommendations set forth by the USDA Dietary Guidelines [34]. Little is known about the food intake, particularly fruits and vegetables, of children on Guam. Pobocik and colleagues [69,70] studied the food intake of over 1000 elementary school-aged children on Guam and reported fruit and vegetable consumption was well below the recommended intake levels [69]. Leon Guerrero and Workman [71] studied health behaviors of adolescents on Guam and reported that only 24.7% of those surveyed reported consumption at least one serving of fruit and/or vegetable, and none reported consuming the recommendations for fruit and vegetable intake set forth by the Dietary Guidelines. Children on Guam are not unlike children from the US mainland. A recent study looking at diet quality by BMI category of US children found that in general, all children’s diets in the United States were poor and in need of improvement, regardless of BMI status, age, sex, race/ethnicity, and poverty-to-income ratio classification [72].

CHamorus and Filipinos were less likely to exclusively breastfeed and tended to breastfeed for shorter durations, compared to other Micronesians and Other ethnic groups; mixed feeding was common in all ethnic groups and associated with earlier weaning as would be expected. No relationship of feeding method with overweight and obesity was detected. Breastfeeding is the biologic norm, important for infant nutrition and healthy growth, and protective for child obesity [73,74,75], although not demonstrated here. Likely other socio-economic (e.g., income) and behavioral factors (e.g., SSB intake) not controlled in this comparison confound the association in this study.

Several studies have shown an association between SSB intake and adverse health outcomes in children, particularly overweight, obesity, unhealthy weight gain, and central adiposity [76,77,78]. Other longitudinal data indicated that children consuming as little as one SSB serving per day were 55% more likely to be OWOB compared to children with limited SSB consumption [79]. Wojcicki and colleagues looked at the effect of SSB consumption on leukocyte telomere length in preschool children [80]. After adjusting for age sex, and BMI, SSB intake among the preschool children was significantly related to shorter leukocyte length. Shorter telomere length has been associated with a number of adverse health outcomes such as type 2 diabetes, stroke, and myocardial infarction because shortened telomeres start the inflammatory cascade through increased production of cytokines, thus adversely impacting cellular health [80]. SSB consumption during early childhood may not lead immediately to OWOB, as there may be an age effect. It may take a number of years of SSB consumption before the development of OWOB or metabolic changes associated with obesity [77,80,81,82]. SSB intake may also be a marker of a food and beverage intake pattern and other lifestyle factors that are associated with obesity.

High intake of SSB is common on Guam, and SSBs are readily available in food stores. In a previous study looking at dietary intake of CHamoru and Filipino adults living on Guam, Leon Guerrero and colleagues [11] found that CHamoru adults reported consuming between 7–9% of food energy from SSBs (7% for women and 9% for men). In this same study [11], SSBs were the second largest contributor to energy intake for CHamoru adults; and third largest contributor to energy intake for Filipino adults.

Like most other islands in the Pacific, Guam relies heavily on imported processed foods. A 2013 survey of processed foods available in the Pacific Islands [83] showed that Guam recorded the highest number of available processed food products compared to the other Pacific islands surveyed, with over 2100 products available to Guam consumers. High intake of SSBs is not only a problem for children affected by OWOB. The child participants surveyed on Guam, whether or not they were affected by OWOB, consumed a high amount of SSBs, putting them at risk for developing adverse cardiometabolic outcomes as they grow older.

There were some limitations to this study. The small sample size of Other Micronesian and especially Filipino children made ethnic group comparisons difficult. Some parents/caregivers, Other Micronesians in particular, spoke English as a second language and had limited English comprehension; making it difficult to comprehend and respond to survey tools. This may have affected accuracy of results. Generalizations are limited due to non-randomized cluster sampling. However, recruitment of children at the community-based settings in Guam communities was designed to ensure appropriate representation by age, sex, ethnicity, and geographic location. Since this study reports cross-sectional data, causal association of OWOB with behavioral or demographic factors cannot be conclusively inferred; longitudinal examination is needed to confirm causality. Despite the limitations, we were able to recruit and assess a large sample of children (*n* = 865) from several communities across the island that were representative of the major ethnic groups on Guam. Anthropometric measurements of children surveyed provided a reliable assessment of OWOB status among children on Guam.

In conclusion, the child participants surveyed on Guam, whether or not they are currently affected by OWOB, are at a high risk for developing OWOB in adulthood, as well as other chronic diseases. Specifically, for OWOB risk factors and CHL behaviors, the majority of children surveyed did not meet recommendations for: sleep duration (59.6%), sedentary screen-time (83.11%), or fruit (58.7%) and vegetable (99.1%) intake and consumed SSB (73.7%). The study found children affected by OWOB in this study, were statistically more likely (*p* = 0.042) to suffer from sleep disturbances, and consumed marginally higher amounts (*p* value = 0.07) of SSB compared to children with healthy weight. The study identified indicators of potentially stressful home situations among participant families that include: high prevalence of the child participants being cared for by a single parent (60.7%), families with three or more children (76.2%), and high prevalence of some level of food insecurity (59.6%) among participant families. These family stressors may also contribute to OWOB among children on Guam. With respect to OWOB prevention guidance, the study found evidence supporting previous studies’ findings that traditional or low acculturation to be protective against obesity [39,59,60]. Healthy cultural behaviors, both unique to and shared across ethnic group(s), should be incorporated in future obesity interventions by highlighting healthy cultural values, practices and traditional foods. The obesogenic factors identified in the children in this study provide a basis for health promotion and obesity prevention guidance for children in Guam.

## Figures and Tables

**Table 1:** Characteristics * of child participants from Guam in the Children’s Healthy Living (CHL) Program by ethnicity.

	Total	CHamoru (*n* = 561)	Other Micronesians (*n* = 223)	Filipino (*n* = 81)	Other (*n* = 10)	*p* Value
**Child Participant**						
Age, years	5.79 ± 0.062	5.80 ± 0.078	5.66 ± 0.123	5.99 ± 0.062	6.24 ± 0.670	
2–5 years old	465 (53.76%)	296 (52.76%)	123 (57.76%)	42 (51.85%)	4 (40%)	0.418
6–8 years old	400 (46.24%)	265 (47.23%)	90 (42.25%)	39 (48.14%)	6 (60%)	
Birthplace						0.0001
Guam	723 (84.46%)	488 (88.09%)	170 (80.19%)	62 (76.54%)	3 (33.33%)	
U.S. Mainland	52 (6.08%)	36 (6.50%)	9 (4.25%)	3 (3.70%)	4 (44.44%)	
Saipan, CNMI	34 (3.97%)	28 (5.05%)	5 (2.36%)	1 (1.24%)	0 (0%)	
Other Islands in Micronesia	28 (3.27%)	0 (0%)	28 (13.21%)	0 (0%)	0 (0%)	
Philippines	15 (1.75%)	0 (0%)	0 (0%)	15 (18.52%)	0 (0%)	
Other	4 (0.47%)	2 (0.36%)	0 (0%)	0 (0%)	2 (22.22%)	
Ever Breastfed?						0.0006
Yes	557 (67.19%)	341 (62.80%)	155 (78.68%)	53 (67.09%)	8 (80%)	
No	272 (32.81%)	202 (37.20%)	42 (21.32%)	26 (32.91%)	2 (20%)	
Child fed both breastmilk & infant formula						0.8223
Yes	443 (54.23%)	291 (54.09%)	102 (53.40%)	46 (58.23%)	4 (44.45%)	
No	374 (45.77%)	247 (45.91%)	89 (46.60%)	33 (41.77%)	5 (55.55%)	
Child age (months) when weaned from breastmilk	9.76 ± 0.438	8.63 ± 0.519	12.48 ± 0.958	8.511 ± 1.165	18.0 ± 3.928	
Average Sleep Duration (h/d)	8.61 ± 0.078	8.86 ± 0.086 ^†^	7.75 ± 0.200	8.99 ± 0.206 ^†^	9.05 ± 0.273	0.0001
TCSQ Sleep Score	6.34 ± 0.205	6.583 ± 0.246	6.011 ± 0.4621	5.231 ± 0.554	8.00 ± 3.386	
Met Sleep Recommendations ^Ƒ^						0.0136
Yes	343 (40.35%)	240 (43.09%)	63 (30.58%)	35 (43.75%)	5 (50%)	
No	507 (59.65%)	317 (56.91%)	143 (69.42%)	45 (65.25%)	5 (50%)	
Average Screen-time (hr/d)	5.29 ± 0.132	5.13 ± 0.154 ^§^	5.45 ± 0.308	6.24 ± 0.425	3.59 ± 0.550	
Met Screen-time Recommendations ^Ω^						0.0069
Yes	124 (16.89%)	82 (16.73%)	39 (22.54%)	3 (4.61%)	0 (0%)	
No	610 (83.11%)	408 (83.27%)	134 (77.46%)	62 (95.39%)	6 (100%)	
Height (cm)	110.56 ± 0.46	109.07 ± 0.59	111.31 ± 1.71	112.98 ± 1.22	114.75 ± 5.03	
Weight (kg)	21.05 ± 0.25	20.78 ± 0.31	21.50 ± 0.54	21.55 ± 0.72	22.4 ± 2.44	
Body Mass Index (kg/m^2^)	16.84 ± 0.11	16.77 ± 0.14	17.09 ± 0.43	16.65 ± 0.27	16.42 ± 0.61	
BMI z-score	0.402 ± 0.039	0.352 ± 0.048 ^†^	0.544 ± 0.08	0.395 ± 0.132	0.189 ± 0.284	0.0397
BMI Categories ^ß^						
Underweight	25 (2.96%)	18 (32.91%)	4 (1.93%)	3 (3.75%)	0 (0%)	0.6945
Healthy weight	595 (70.49%)	392 (71.66%)	144 (69.56%)	51 (63.75%)	8 (80%)	0.4523
Overweight, % (≥85th%ile)	113 (13.39%)	77 (14.08%)	21 (10.14%)	14 (17.50%)	1 (10%)	0.3385
Obesity, % (≥95th%ile)	111 (13.15%)	60 (10.97%)	38 (18.36%)	12 (15.0%)	1 (10%)	0.0314
OWOB by Age						
2–5 years old	107 (23.94%)	59 (20.85%)	32 (26.89%)	16 (39.02%)	0 (0%)	0.0378
6–8 years old	117 (29.47%)	78 (29.54%)	27 (30.68%)	10 (25.64%)	2 (33.33%)	0.9441
Waist Circumference (cm)	55.07 ± 0.30	54.76 ± 0.38	55.44 ± 0.58	56.104 ± 0.95	55.64 ± 2.50	
Abdominal Obesity ^€^						0.6782
Yes	76 (8.94%)	48 (8.77%)	17 (7.98%)	10 (12.50%)	1 (10%)	
No	774 (91.06%)	499 (91.23%)	196 (92.02%)	70 (87.5%)	9 (90%)	
Consumed SSB?						0.0001
Yes	497 (74.74%)	361 (81.49%)	95 (61.29%)	36 (59.01%)	5 (50%)	
No	168 (25.26%)	82 (18.51%)	60 (38.71%)	25 (40.99%)	5 (50%)	
Beverage Consumption ^‡,¶^						
Water (c/d)	1.47 ± 0.048	1.306 ± 0.45	1.298 ± 0.66	1.401 ± 0.105	1.522 ± 0.429	
Milk (c/d)	1.241 ± 0.024	1.280 ± 0.029 ^†,§^	1.028 ± 0.045	1.454 ± 0.081 ^†^	1.660 ± 0.139	
Sugar-sweet drinks (c/d)	0.845 ± 0.029	0.958 ± 0.036 ^†,§,‡^	0.560 ± 0.050 ^Ø^	0.727 ± 0.101	1.095 ± 0.269	
Fruit Consumption ^‡^ (c/d)	0.882 ± 0.021	0.88 ± 0.025	0.846 ± 0.045	0.956 ± 0.080	1.153 ± 0.240	
Vegetable Consumption ^‡,¶^ (c/d)	0.609 ± 0.013	0.648 ± 0.015	0.472 ± 0.026	0.651 ± 0.041	0.850 ± 0.139	
Met Recommendation for:						
Fruit Intake	257 (41.35%)	182 (41.08%)	63 (40.65%)	27 (44.26%)	3 (50%)	0.9312
Vegetable Intake	6 (0.90%)	5 (1.13%)	1 (0.65%)	0 (0%)	0 (0%)	0.8064
**Parent/Caregiver/Household**						
Annual Family Income Level						0.0001
<$20,000	348 (57.91%)	220 (54.18%)	92 (82.88%)	32 (43.24%)	4 (40%)	
$20,000–$34,999	182 (14.81%)	59 (14.53%)	9 (8.11%)	17 (22.97%)	4 (40%)	
$35,000–$59,999	93 (15.47%)	71 (17.49%)	5 (4.51%)	17 (22.97%)	0 (0%)	
>$60,000	71 (11.81%)	56 (13.80%)	5 (7.20%)	8 (10.82%)	2 (20%)	
Receiving any Food Assistance						0.0001
Yes	676 (80.38%)	447 (81.57%)	176 (86.70%)	47 (58.75%)	6 (60%)	
No	165 (19.62%)	101 (18.43%)	27 (13.30%)	33 (41.25%)	4 (40%)	
Type of Food Assistance						
SNAP ^∂^	585 (67.85%)	401 (71.61%)	146 (69.19%)	33 (40.74%)	5 (50%)	0.0001
Local Food Bank	101 (11.72%)	69 (12.32%)	28 (13.27%)	4 (4.94%)	0 (0%)	0.1319
WIC ^∞^	274 (31.79%)	162 (28.93%)	86 (40.76%)	22 (27.16%)	4 (40%)	0.0114
Free School Lunch/Breakfast	248 (28.77%)	202 (36.07%)	33 (15.64%)	10 (12.35%)	3 (30%)	0.0001
Food Insecurity ^δ^						0.0001
Always/most times	127 (16.82%)	86 (17.20%)	32 (18.71%)	7 (9.46%)	2 (20%)	
Sometimes	262 (34.70%)	128 (25.6%)	96 (56.16%)	35 (47.29%)	3 (30%)	
Seldom/never	366 (48.48%)	286 (57.20%)	43 (25.15%)	32 (43.24%)	5 (50%)	
Parent/Caregiver Education						0.0001
<12th Grade	281 (32.48%)	184 (32.80%)	79 (37.08%)	17 (20.98%)	1 (10%)	
12th Grade/GED	351 (40.58%)	237 (42.24%)	97 (45.54%)	13 (16.05%)	4 (40%)	
Some college or higher	233 (26.94%)	140 (24.96%)	37 (17.38%)	32 (62.97%)	5 (50%)	
Parent Marital Status						0.0001
Married	340 (39.31%)	190 (33.89%)	90 (42.25%)	55 (67.90%)	5 (50%)	
Not Married	525 (60.69%)	371 (66.13%)	123 (57.75%)	26 (32.10%)	5 (50%)	
Number Children in Household	4.19 ± 0.077	4.29 ± 0.096 ^§^	4.35 ± 0.164 ^§^	3.14 ± 0.151	3.4 ± 0.306	0.0001
Household Size						0.008
1–2 children	205 (23.78%)	127 (22.72%)	48 (22.64%)	29 (35.80%)	1 (1%)	
3–4 children	341 (39.56%)	220 (39.36%)	75 (35.38%)	39 (48.15%)	7 (70%)	
5 or more children	316 (36.66%)	212 (37.92%)	89 (41.98%)	13 (16.05%)	2 (20%)	
Acculturation ^γ^						0.0565
Integrated	572 (79.89%)	388 (80.15%)	123 (75.00%)	55 (88.71%)	6 (100%)	
Traditional	101 (14.11%)	74 (15.29%)	24 (14.63%)	3 (4.84%)	0	
Assimilated	17 (2.37%)	7 (1.45%)	7 (4.27%)	3 (4.83%)	0	
Marginalized	26 (3.63%)	15 (3.10%)	10 (6.10%)	1 (1.61%)	0	

* Frequency number (%), ^¶^ Mean ± standard error, ^‡^ Consumption in cups/day, weighted for weekday/weekend days and adjusted for within person variance, ^†^ Significantly (*p* < 0.05) different from Other Micronesians ^§^ Significantly (*p* < 0.05) different from Filipino, ^Ø^ Significantly (*p* < 0.05) different from Other, ^Ƒ^ Sleep Recommendations: at least 11 h/d for 2-year-olds, at least 10 h/d for 3 to 5 year-olds, and at least 9 h/d for 6 to 10 year-olds, ^Ω^ Screen-time Recommendation of 2 h/d or less, ^ß^ BMI percentiles calculated according to CDC reference data and categories assigned as: underweight (<5th percentile), healthy weight (5th–84th percentile), overweight (85th–94th percentile), and obese (95th percentile), ^€^ Abdominal obesity defined as waist circumference greater than the International Diabetes Federation cut-point (>90.2 percentile for age and sex), ^∂^ USDA Supplemental Nutrition Assistance Program (SNAP), ^∞^ USDA Supplemental Nutrition Assistance Program for Women, Infants, and Children (WIC), ^δ^ Question from USDA Core Food Security Module “In the past 12 months how often does money for food run out by the end of the month? ^γ^ What category of acculturation does family consider themselves?

**Table 2:** Characteristics of child participants from Guam in the Children’s Healthy Living (CHL) Program stratified by BMI status.

Participant & Family Characteristics	Total Mean ± SE or *n*(%)	Healthy Weight * Mean ± SE or *n* (%)	OWOB * Mean ± SE or *n* (%)	*p* Value
Age				0.069
2–5 years old	437 (53.2%)	329 (55.1%)	108 (48%)	
6–8 years old	385 (46.8%)	268 (44.9%)	117 (52%)	
Sex				0.917
Boys	425 (51.7%)	308 (51.6%)	117 (52.0%)	
Girls	397 (48.3%)	289 (48.4%)	108 (48.0%)	
Child Ethnicity				0.455
CHamoru	528 (64.55%)	392 (65.88%)	137 (61.16%)	
Other Micronesians	203 (24.82%)	144 (24.20%)	59 (26.34%)	
Filipino	77 (9.41%)	51 (8.57%)	26 (11.61%)	
Other	10 (1.22%)	8 (1.34%)	2 (0.89%)	
Annual Family Income Level				0.473
<$20,000	331 (57.6%)	234 (56.9%)	97 (59.1%)	
$20,000–$34,999	86 (15%)	63 (15.3%)	23 (14%)	
$35,000–$59,999	89 (15.5%)	60 (14.6%)	29 (17.7%)	
>$60,000	69 (12%)	54 (13.1%)	15 (9.1%)	
Receiving any Food Assistance				0.768
Yes	645 (78.5%)	470 (78.7%)	175 (77.8)	
No	177 (21.5%)	127 (21.3%)	50 (22.2%)	
Type of Food Assistance				
SNAP	556 (67.9%)	408 (68.7%)	148 (65.8%)	0.426
Local Food Bank	99 (12.15%)	72 (72.7%)	27 (27.3%)	0.977
WIC	258 (31.5%)	196 (33.0%)	62 (27.6%)	0.135
Free School Lunch/Breakfast	238 (29.1%)	169 (28.5%)	69 (30.7%)	0.533
Food Insecurity				0.566
Always/most times	120 (16.7%)	88 (16.8%)	32 (16.3%)	
Sometimes	253 (35.2%)	178 (34%)	75 (38.3%)	
Seldom/never	346 (48.1%)	257 (49.1%)	89 (45.4%)	
Parent/Caregiver Education				0.052
<12th Grade	269 (32.7%)	207 (34.7%)	62 (27.6%)	
12th Grade/GED	331 (40.3%)	241 (40.4%)	90 (40%)	
Some college or higher	222 (27%%)	149 (25%)	73 (32.4%)	
Parent Marital Status				0.06
Married	326 (39.7%)	225 (37.7%)	101 (44.9%)	
Not Married	496 (60.3%)	372 (62.3%)	124 (55.1%)	
Number Children in Household	4.19 ± 0.077	4.19 ± 0.094	4.22 ± 0.152	0.838
Household Size				0.491
1–2 children	197 (24.1%)	145 (24.4%)	52 (23.1%)	
3–4 children	319 (38.9%)	224 (37.7%)	95 (42.2%)	
5 or more children	303 (37%)	225 (37.9%)	78 (34.7%)	
Birthplace				0.0070
Guam	685 (84.46%)	505 (85.45%)	180 (81.45%)	
U.S. Mainland	52 (6.41%)	37 (6.26%)	15 (6.79%)	
Saipan, CNMI	31 (3.82%)	20 (3.38%)	13 (5.88%)	
Other Islands in Micronesia	25 (3.08%)	21 (3.55%)	4 (1.81%)	
Philippines	13 (1.60%)	8 (1.35%)	5 (2.26%)	
Other	4 (0.49%)	1 (0.17%)	4 (1.82%)	
Acculturation				0.2913
Integrated	542 (80.30%)	394 (79.60%)	148 (82.22%)	
Traditional	94 (13.78%)	72 (14.54%)	21 (12.14%)	
Assimilated	17 (2.52%)	10 (2.02%)	7 (3.89%)	
Marginalized	23 (3.41%)	19 (3.84%)	4 (2.22%)	
Ever Breastfed?				0.2140
Yes	529 (64.59%)	373 (66.13%)	156 (70.59%)	
No	256 (35.41%)	191 (33.87%)	65 (29.41%)	
Exclusively Breastfed?				0.5655
Yes	97 (13.13%)	68 (12.69%)	29 (14.29%)	
No	642 (86.87%)	468 (87.13%)	174 (85.71%)	
Child age (mos) when weaned from breastmilk	9.76 ± 0.438 (*n* = 495)	10.22 ± 0.542	9.23 ± 0.844	0.3248
Child fed both breastmilk & infant formula				0.5135
Yes	421 (54.39%)	300 (53.67%)	121(56.28%)	
No	353 (45.61%)	259 (44.33%)	94 (43.72%)	
Average Sleep Duration (h/d)	8.62 ± 0.08	8.64 ± 0.096	8.59 ± 0.142	0.7122
TCSQ Sleep Score	6.34 ± 0.205	6.55 ± 0.257	5.59 ± 0.341	0.0421
Met Sleep Recommendations				0.725
Yes	327 (40.4%)	236 (40%)	91 (41.4%)	
No	483 (59.6%)	354 (60%)	129 (58.6%)	
Average Screen-time (h/d)	5.26 ± 0.132	5.15 ± 0.150	5.55 ± 0.270	0.1743
Met Screen-time Recommendations				0.444
Yes	117 (16.8%)	82 (16.2%)	35 (18.6%)	
No	578 (83.2%)	425 (83.8%)	153 (81.4%)	
Height (cm)	110.52 ± 0.47	108.71 ± 0.528	115.34 ± 0.912	0.0001
Weight (kg)	21.26 ± 0.26	18.77 ± 0.185	27.91 ± 0.635	0.0001
BMI, kg/m^2^	16.95 ± 0.11	15.64 ± 0.038	20.45 ± 0.280	0.0001
BMI z-score	0.485 ± 0.036	0.007 ± 0.026	1.79 ± 0.045	0.0001
Waist Circumference (cm)	55.07 ± 0.457	51.93 ±0.188	64.40 ± 0.707	0.0001
Abdominal Obesity				0.0001
Yes	75 (9.2%)	0 (0%)	75 (33.60%)	
No	741 (90.8%)	591 (100%)	148 (66.40%)	
Beverage Consumption ^‡^				
Water (c/d)	1.31 ± 0.035	1.32 ± 0.043	1.28 ± 0.070	0.5793
Milk (c/d)	1.24 ± 0.24	1.25 ± 0.028	1.21 ± 0.048	0.5994
Sugar-sweetened drinks (c/d)	0.84 ± 0.03	0.81 ± 0.34	0.93 ± 0.061	0.0676
Vegetable Intake ^‡^ (c/d)	0.61 ± 0.013	0.60 ± 0.015	0.63 ± 0.027	0.2535
Fruit Intake ^‡^ (c/d)	0.88 ± 0.021	0.87 ± 0.025	0.89 ± 0.040	0.7324
Met Recommendation for:				
Fruit Intake	257 (41.35%)	185 (39.96%)	79 (46.20%)	0.1571
Vegetable Intake	6 (0.90%)	4 (0.86%)	2 (1.17%)	0.7242

* Healthy weight defined as BMI between 5th–84th percentile for age and sex; ‘OWOB’ defined as BMI greater than 85th percentile for age and sex, ^‡^ Consumption in cups/day, weighted for weekday/weekend days and adjusted for within person variance.

**Table 3:** Prevalence and Odds of OWOB among child participants from Guam in the Children’s Healthy Living (CHL) Program.

Variable	*n*	% OWOB ^†^ (95% CI)	Adjusted OR ^††^ (95% CI)
Overall	822	27.4 (24.3–30.4)	
Child Characteristics			
Sex			
Males	425	27.5 (23.3–31.8)	Referent
Females	397	27.2 (22.8–31.6)	1.001 (0.734–1.366)
Age			
2–5 years	437	24.7 (20.7–28.8)	Referent
6–8 years	385	30.4 (25.8–35.0)	1.337 (0.982–1.821)
Ethnicity			
CHamoru	531	26.0 (22.3–29.7)	Referent
Other Micronesians	204	28.9 (22.7–35.2)	1.186 (0.824–1.706)
Filipino	77	33.8 (23.0–44.6)	1.454 (0.870–2.430)
Other	10	20.0 (−10.2–50.2)	0.693 (0.144–3.329)
Child was breastfed?			
Yes	530	29.4 (25.5–33.3)	1.212 (0.861–1.708)
No	258	25.6 (20.2–30.9)	Referent
Child met sleep standard for his/her age group?			
Yes	327	27.8 (23.0–32.7)	0.994 (0.717–1.378)
No	483	26.7 (22.8–30.7)	Referent
Child met recommendation for screen-time of ≤ 2 h/day?			
Yes	117	29.9 (21.5–38.3)	1.184 (0.757–1.851)
No	578	26.5 (22.9–30.1)	Referent
Child met recommendation for vegetable intake for his/her age group?			
Yes	6	33.3 (−20.9–87.5)	1.342 (0.236–7.649)
No	628	26.9 (23.4–30.4)	Referent
Child met recommendation for fruit intake for his/her age group?			
Yes	264	29.9 (24.4–35.5)	1.274 (0.89–1.823)
No	370	24.9 (20.4–29.3)	Referent
SSB beverage intake ^‡^ (cups/day)			
(zero intake—referent)	160	21.3 (14.8–27.7)	Referent
Tertile 1 (≤0.42)	54	33.3 (20.4–46.3)	2.064 (1.024–4.160)
Tertile 2 (0.42–1.09)	208	26.4 (20.4–32.5)	1.495 (0.90–2.485)
Tertile 3 (≥1.09)	212	30.2 (24.0–36.4)	1.824 (1.106–3.007)
*p*-value for trend			0.022
Water beverage intake ^‡^ (cups/day)			
(zero intake—referent)	26	30.8 (11.8–49.8)	Referent
Tertile 1 (≤0.92)	195	26.2 (19.9–32.4)	0.827 (0.333–2.057)
Tertile 2 (0.92–1.62)	201	28.4 (22.1–34.6)	0.965 (0.388–2.401)
Tertile 3 (≥1.62)	212	25.9 (20.0–31.9)	0.819 (0.330–2.033)
*p*-value for trend			0.597
Parent/Caregiver/Household Characteristics			
Education Level			
Less than 12th grade (use this as reference)	269	23.1 (18.0–28.1)	Referent
12th grade/GED or higher	553	29.5 (25.7–33.3)	1.415 (1.004–1.994)
Marital Status			
Married	326	31.0 (25.9–36.0)	Referent
Not married	496	25.0 (21.2–28.8)	0.776 (0.564–1.067)
Annual household income			
<$20,000	331	29.3 (24.4–34.2)	Referent
$20,000–$34,999	86	26.7 (17.2–36.3)	0.867 (0.501–1.502)
$35,000–$59,999	89	32.6 (22.7–42.5)	1.121 (0.664–1.892)
$60,000+	69	21.7 (11.8–31.7)	0.600 (0.315–1.143)
Food Insecurity			
Yes	120	26.7 (18.6–34.7)	0.982 (0.627–1.539)
No	599	27.4 (23.8–31.0)	Referent
Receiving any food assistance			
Yes	645	27.1 (23.4–30.6)	1.027 (0.699–1.509)
No	177	28.3 (21.6–35.0)	Referent
Number of children in household			
1–2 children	197	26.4 (20.2–32.6)	Referent
3–4 children	319	29.8 (24.7–34.8)	1.18 (0.789–1.766)
5 or more children	303	25.7 (20.8–30.7)	0.948 (0.622–1.446)
Acculturation—Family considers themselves ‘integrated’ into culture			
Yes	545	27.3 (23.6–31.1)	1.162 (0.747–1.81)
No	134	24.6 (17.2–32.0)	Referent

^†^‘ Underweight’ children excluded from analysis, ^††^ Adjusted for clustering by community, age, sex, and ethnicity, ^‡^ Consumption in cups/day, weighted for weekday/weekend days and adjusted for within person variance.
